# Outcomes and adverse events of pre- and extensively drug-resistant tuberculosis patients in Kinshasa, Democratique Republic of the Congo: A retrospective cohort study

**DOI:** 10.1371/journal.pone.0236264

**Published:** 2020-08-04

**Authors:** Innocent Murhula Kashongwe, Fina Mawete, Leopoldine Mbulula, Don Jean Nsuela, Luc Losenga, Nicole Anshambi, Murielle Aloni, Michel Kaswa, Jean Marie Ntumba Kayembe, Pierre Umba, Francois Bompeka Lepira, Zacharie Munogolo Kashongwe

**Affiliations:** 1 Kinshasa University Hospital, Internal Medicine, Pulmonology Unit, Kinshasa, Democratic Republic of the Congo; 2 Drug Resistant Tuberculosis Unit ‘Centre d’excellence Damien’, Damian Foundation, Kinshasa, Democratic Republic of the Congo; 3 National Tuberculosis Program of the Democratic Republic of the Congo, Kinshasa, Democratic Republic of the Congo; 4 Provincial Coordination for Tuberculosis Control, Kinshasa, Democratic Republic of the Congo; 5 National Laboratory of Mycobacteria, Kinshasa, Democratic Republic of the Congo; University of Cape Town Institute of Infectious Disease and Molecular Medicine, SOUTH AFRICA

## Abstract

**Background:**

Extensively drug-resistant tuberculosis (XDR TB) is a very serious form of tuberculosis that is burdened with a heavy mortality toll, especially before the advent of new TB drugs. The Democratic Republic of the Congo (DRC) is among the countries most affected by this new epidemic.

**Methods:**

A retrospective analysis was performed of the records of all patients with pre- and extensively drug-resistant tuberculosis hospitalized from January 1, 2015 to December 31, 2017 and monitored for at least 6 months to one year after the end of their treatment in Kinshasa; an individualized therapeutic regimen with bedaquiline for 20 months was built for each patient. The adverse effects were systematically monitored.

**Results:**

Of the 40 laboratory-confirmed patients, 32 (80%) patients started treatment, including 29 preXRB and 3 XDR TB patients. In the eligible group, 3 patients (9.4%) had HIV-TB coinfections. The therapeutic success rate was 53.2%, and the mortality rate was 46.8% (15/32); there were no relapses, failures or losses to follow-up. All coinfected HIV–TB patients died during treatment. The cumulative patient survival rate was 62.5% at 3 months, 53.1% at 6 months and 53.1% at 20 months. The most common adverse events were vomiting, Skin rash, anemia and peripheral neuropathy.

**Conclusion:**

The new anti-tuberculosis drugs are a real hope for the management of Drug Resistant tuberculosis patient and other new therapeutic combinations may improve favorable outcomes.

## Introduction

The global epidemiology of drug resistance in tuberculosis has worsened over the past 20 years, particularly with the emergence of multidrug-resistant (MDR)-TB and extensively drug-resistant (XDR)-TB [1–4]. Extensively drug-resistant TB is defined as MDR-TB plus resistance to at least one drug fluoroquinolones and second-line injectable agents (amikacin, capreomycin or kanamycin) [1]. The injectable drugs are less used due to high number of side effects, poor efficacity and discomfort for patients administration [5, 6].

Of all MDR TB strains, 8.5% meet the extensively drug-resistant (XDR) definitions [1, 2, 7]. A WHO TB report in 2015 showed that among 580,000 eligible patients for multidrug-resistant tuberculosis (MDR-TB) treatment, only 20% received an appropriate drug regimen, and 250, 000 patients died [1].

By the end of 2018, at least one case of XDR-TB had been reported by 131 WHO Member States [1]; three countries accounted for almost half of the world’s cases of MDR/RR-TB: India (24%), China (13%), and the Russian Federation (10%). The Democratic Republic of the Congo (DRC) is one of the 30 countries that bear the burden of TB in the world; it is among the 27 leading countries for the drug-resistant form and is among the top 4 countries in Africa, along with South Africa, Nigeria and Ethiopia [1].

Until 2015, the treatment outcome data show a low treatment success rates (34%) for extensively drug-resistant Tuberculosis patient TB (XDR-TB) [8] worldwide. The rate of death among pre-XDR and XDR TB patients in DRC in 2015 reached, 90%, may be, related to unavailability of relevant appropriate treatment alternatives [9, 10].

Apart from being a great challenge, the treatment of MDR- and XDR-TB requires regular assessments. New, more active, and cheaper regimens with fewer side effects are urgently needed to reduce the burden of the disease. The long duration of treatment also needs to be addressed to improve patient adherence [3, 7, 11]. The recent arrival of novel therapeutics recommended by the WHO, such as bedaquiline (Bdq) in June 2013 and delamanid in October 2014, led to great optimism around the world [12, 13]. This news drug fits the need for ‘individualized, all oral regimens, to be used all around the worlds today [14].

However, most of the global use of bedaquiline occurred in two countries, South Africa and Russia, for several reasons (national preference, financial and administrative accessibility) [15].

The combination of the old antituberculosis drugs (such as linezolid, clofazimine and cycloserine) with the new drugs (such as delamanid or pretomanid) is currently a therapeutic hope for the management of these patients [3, 16–21].

Since 2015, the DRC National Tuberculosis Program has approved the use of bedaquiline for the treatment of extensively drug-resistant tuberculosis [9, 10].

However, the effectiveness of the regimens for drug-resistant tuberculosis requires the inclusion of a sufficient number of effective drugs [12]. The main difficulty is the identification of at least four active drugs to design an effective regimen [12, 18, 22, 23]. It is a significant challenge for clinicians worldwide; many of these clinicians are forced to make therapy decisions without any drug susceptibility testing (DST) information [24, 25]. The rapid evolution of knowledge has allowed the WHO to modify the classification of second-line antituberculosis drugs and principles of their combinations twice since 2015 [5, 12, 22, 23].

The aim of the present study was to retrospectively evaluate the effectiveness, safety and tolerability of bedaquiline-containing regimens in pre-XDR and XDR-TB patients in Kinshasa, capital of the DRC.

## Materials and methods

### Ethics statement

Ethical approval for the retrospective collection of clinical data was obtained by the National Tuberculosis Program of the DR Congo (PNLT/01/JTN/MKK/525/2019). Data for analysis were collected anonymously; all patient records were coded by a number designated based on the date of treatment initiation; the number one was the first patient to be treated with bedaquiline containing treatment.

### Study design and participants

This is a retrospective study of all XDR TB and pre-XDR TB patients records who started their treatment with a bedaquiline-based regimen between January 1, 2015 and December 31, 2017 (with follow-up until December 2019).

The confirmation of the diagnosis was made by the National Tuberculosis Laboratory of Mycobacteria of the DR Congo; the phenotypic drug susceptibility test (culture) and the molecular method (Xpert® MTB/RIF testing, Cepheid, Sunnyvale, CA, USA with the line probe assay (LPA) for first-line drug susceptibility and second-line drug susceptibility were performed.

Hospitalization was mandatory for all patients at the Specialized Center for the Management of Complications of Multidrug-Resistant Tuberculosis of Kinshasa called the ‘Centre d’Excellence Damien’ (CEDA). During hospitalization, all patients were evaluated daily by a general practitioner, stable patients were evaluated twice per week by a specialist in tuberculosis and respiratory diseases, and sick patients were evaluated daily (Annex 1). The DR TB regimen was modified by a doctor if severe adverse events were observed [26]. A standardized classification of the severity of the adverse events (AEs) was used. The classification for grading AE severity in adults is based on the scale of ‘the Agence Nationale de recherche sur le SIDA’ [20]. The adverse events were attributed to bedaquiline if the QT interval corrected using the Bazett formula (QTcB) was >500 ms without another possible reason.

Ambulatory management in the health care center was provided for stable patients with three consecutive negative sputum smears or stable patients in the continuation phase. The duration of hospitalization was variable depending on the management of the side effects and clinical follow-up of the patients. Globally, paraclinical follow-up was performed monthly or in cases of emergency (Annex 1). To ensure good compliance after hospitalization, a financial support for transport was given to all patient during the follow up.

### Pre-XDR/XDR-TB treatment and monitoring

The tuberculosis (TB) regimens used during this study were built according to the WHO guidelines in effect at the time of treatment (2015–2017), before the current 2019 recommendations [27–29].

An individualized TB treatment containing bedaquiline was discussed by the National Therapeutic Committee team of the National Tuberculosis Program (NTP) of the DRC. The choice of drug depended on the history previous drugs used, the drug susceptibility testing (DST) if available and the second-line drugs available in the country. Since 2018, a significant change in the classification of second-line anti-TB drugs has taken place, and the principle of a completely oral treatment strategy has significantly reduced the use of aminoglycosides, which were poorly tolerated [30, 31].

Globally, PreXDR-TB and XDR-TB patients are treated with a bedaquiline regimen that could include other second line drugs as needed [injectables such as amikacin (Am) or kanamycin, (km) if susceptible, levofloxacin (Lfx), linezolid (Lzd), clofazimine (Cfz), para-amino salicylic acid (PAS), cycloserine (Cs), high-dose isoniazid (Hhd), pyrazinamide (Z), and prothionamide (Pto)].

bedaquiline was administered at the recommended dose of 400 mg once a day for 14 days, then at 200 mg three times a week for 22 weeks. It was prescribed under programmatic conditions, expanded access and compassionate use, but not under the experimental protocols of clinical trials.

Adverse events were evaluated every day; these events was attributed to bedaquiline or to another particular drug by a doctor (general practitioner, specialist and pharmacovigilance team), the National Committee team of the National Tuberculosis Program and sometimes international experts without documented levels of ascertainment of the causal association.

### Case definition

Case definitions and outcomes were based on the WHO guidelines [22, 31].

### Data variables and source

A data collection sheet was established to collect the epidemiological data (i.e., age; sex, residence), clinical data (i.e., cardiac and thyroid disorders, HIV infection status, previous TB diagnosis and treatment), radiological findings, sputum smear and culture results at baseline, each month during treatment and 12 months after the end of the treatment, the WHO treatment outcomes and the duration of hospital stay.

### Statistical analysis

The data were analyzed using Statistical Package for Social Sciences (Chicago) software for Windows version 24 and XLSTAT 2019. Statistical data processing consisted of calculating the averages, medians, extremes and discrepancies. The types of quantitative variables and the proportions of qualitative variables were also recorded. The results are presented in the form of graphs and tables. The chi-square test compared the proportions, and the Student's t-test compared the averages. Patient survival was described using Kaplan-Meier curves, and the log-rank test compared different curves. The predictors of mortality were investigated by the Cox (Cox proportional hazards) regression test in univariate and multivariate analyses, and the calculated HRs were used to estimate the degree of the associations between mortality and the independent variables. A p <0.05 was the statistical significance threshold.

## Results

### General characteristics

Thirty-two patients were included in this study: two patients in 2015 and fifteen patients respectively in 2016 and 2017. There were 18 men (56.3%) and 14 women (43.7%). Their average age was 32.4 ± 13.4 years (range: 15–68 years). The majority of patients (72%) had been previously treated for multidrug-resistant tuberculosis, and 41% of them had extensive radiological lesions at the baseline investigations. The majority of patients came from the Funa district, one of the districts of Kinshasa with a high population density. The incidence of HIV-TB coinfections was low in this study, with only 3 patients (9.4%) having coinfections. Three patients had diabetes mellitus (9.4%); of these patients, one was diagnosed with diabetes during the current TB treatment. The nutritional status was poor in 66% of patients. According to the category of resistance, we could distinguish twenty-nine preXDR-TB patients, including twenty-five (78.1%) pre XDR with Fluoroquinolone resistance [Pre-XDR _FQ_], four (12,5%) pre XDR with second line injectable resistance [Pre-XDR _SLID(Am/Km)_] and three XDR TB patients.

The mean duration of hospitalization was 300.8 ± 270.5 days (165 days), with extremes of five and 610 days. All general characteristic of the study population are summarized in the Table 1.

**Table 1:** General characteristics and available drug susceptibility testing results.

**Characteristics**	***All* (n = 32)**	**%**
**Age**		
Age±SD and median [IQR]	32.4±13.4	41.5 [15–68]
<40 years	26	81.3
≥40 years	6	18.8
**Sex**		
Male	18	56.3
Female	14	43.7
**Residence**		
Lukunga	5	1.6
Mont Amba	5	15.6
Funa	14	43.8
Tshangu	8	25.0
**Previous MDR TB treatment Lung Lesions**	23	71.9
< 50%	19	59.4
≥50%	13	40.6
**Diabetes mellitus**	2	6.3
**HIV**	3	9.4
**BMI,** kg/m^2^, SD, median [IQR]	16.9±3.5	18.5 [16.4–20.6]
<18.5	21	65.6
18.5–24.9	11	34.4
**Gen Xpert** RR TB	31*	97
**Available Phenotypic First-Line DST Results**		
E	1	3.1
RHE	1	3.1
RHES	12	37.5
RH	12	37.5
**Resistance Category**		
Pre-XDR _SLID(Am/Km)_	4	12.5
Pre-XDR _FQ_	25	78.1
XDR	3	9.4

*one patient had an extrapulmonary preXDR TB

### Outcomes

Seventeen patients (53.2%) completed their treatment successfully. Fifteen patients died (46.8%) during treatment; no deaths occurred in 2015, eleven patients (11/15) died in 2016, and four patients (4/15) died in 2017. The circumstances of death were variable (Table 2). There were no failures, no loss to follow-up and no relapses between 6 months to 1 year post-treatment at the time of the analysis. The Table 2 summarized the circumstances of death.

**Table 2:** Circumstances of death.

Circumstances of death	n
Heart rhythm disorder • One case of atrial and ventricular extrasystole • One case of persistent flat T waves despite preventive therapy with potassium and magnesium	2
Chronic respiratory insufficiency	2
Sudden death One of had a very high QTc without potassium disorders or another cause	3
Gastrointestinal bleeding	2
Septic shock	4
Heart failure	1
Severe depression	1

### Pre-XDR/XDR treatment and adverse events (AEs)

Several bedaquiline-containing treatments were applied. The most common side effects were vomiting (46,9%), skin rash (46,9%), peripheral neuropathy (46,9%) and anemia (43,8%). Serious AEs were reported in 9 patients. The full list of side effects is shown in the Table 3.

**Table 3:** Adverse events (AE).

**Adverse Events**	**All n = 32**	**ANRS AE D1**	**Severity D2**	**D3**	**D4**
Vomiting	15 (46,9)	2 (13,3)	13 (86,7)	0 (0)	0 (0)
Skin Rash	15 (46,9)	3 (20,0)	12 (80,0)	0 (0)	0 (0)
Peripheral Neuropathy	15 (46,9)	6 (40,0)	7 (46,7)	2 (13,3)	0 (0)
Anemia	14 (43,8)	4 (28,6)	3 (21,4)	4 (28,6)	3 (21,4)
Hyperuricemia	10 (31,3)	7 (70,0)	3 (30,0)	0 (0,0)	0 (0,0)
Diarrhea	9 (28,1)	4 (44,4)	5 (55,6)	0 (0)	0 (0)
Nausea	7 (21,9)	2 (28,6)	5 (71,4)	0 (0)	0 (0,0)
Hypokalemia	7 (21,9)	1 (14,3)	2 (28,6)	3 (42,9)	1 (14,3)
Abdominal pain	5 (15,6)	2 (40,0)	3 (60,0)	0 (0)	0 (0)
Otovestibular toxicity	5 (15,6)	2 (40,0)	1 (20,0)	2 (40,0)	0 (0)
Depression	4 (12,5)	0(0)	0 (0,0)	1 (25,0)	3 (75,0)
Fear of heights	3 (9,4)	3 (100)	0 (0,0)	0 (0)	0 (0)
Thrombocytopenia	3 (9,4)	1 (33,3)	1 (33,3)	0 (0)	1 (33,3)
QT prolongation	3 (9,4)	0 (0)	3 (100,0)	0 (0)	0 (0)
Optic neuropathy	2 (6,3)	0 (0)	0 (0,0)	1 (50,0)	1 (50,0)
Blurred vision	2 (6,3)	2 (100)	0 (0,0)	0 (0)	0 (0)
Gastritis	1 (3,1)	1 (100)	0 (0,0)	0 (0)	0 (0)
Onychomycosis	1 (3,1)	0 (0)	1 (100,0)	0 (0)	0 (0)
Hepatotoxicity	1 (3,1)	0 (0)	0 (0)	1 (3,1)	0 (0)
Hyperglycemia	1 (3.1)	0 (0)	0 (0)	1 (3,1)	0 (0)

D1 = mild or transient discomfort without limitation of normal daily activities; no medical intervention or corrective treatment required. D2 = moderate limitation of normal daily activities, with minimal intervention or corrective treatment required. D3 = severe limitation or reduction of daily activities; intervention or treatment corrective are mandatory; and D4 = removal of suspected drugs, discontinuation or stop treatment.

Four patients (12.5%) had a therapeutic modification (such as discontinuation or removal of linezolid, bedaquiline, or aminoglycoside), and these drugs were replaced with another drug if necessary. The majority of deceased patients had a high dose of isoniazid in their therapeutic regimen. All treatment is given in the Table 4.

**Table 4:** Bedaquiline-containing treatments according to the outcomes.

**DR treatment used**	**Alive n = 17**	**Died n = 15**
4 Am, 6 Bdq Hhd, PAS, Lzd, Cfz, Z/14 Lzd, PAS, Cfz, Z	3(17,6)	8(53,3)
4 Am, 6Bdq Hhd, Lfx, Lzd, Cfz, Z/14 Lfx, Lzd, Cfz, Z	1(5,9)	3(20,0)
6 Lfx, Bdq, Hhd, PAS, Lzd, Cfz, Z/14 Lfx, Lzd, PAS, Cfz, Z	0(0,0)	2(13,3)
4 Am 6 Bdq, PAS, Lzd, Cfz, Z/14 Pas, Lzd, Cfz, Z	6(35,3)	1(6,7)
6 Km, Bdq, Hhd, Pas, Lzd, Cfz, Z/14 Lzd, PAS, Cfz, Z	0(0,0)	1(6,7)
4 Am, 6 Bdq, PAS, Lzd, Cfz, Z/14 Lzd, PAS, Cfz, Z	2(11,8)	0(0,0)
6Am, Bdq, Pto, PAS, Lzd, Cs, Z/14 PAS, Lzd, Cs, Z	2(11,8)	0(0,0)
6 Km, Bdq, Hhd, PAS, Lzd, Cfz, Z/14 Lzd, PAS, Cfz, Z	2(11,8)	0(0,0)
6 Mpm-clv, Bdq, PAS, Lzd, Cfz, Z/6 Bdq, PAS, Lzd, Cfz, Z/8 PAS, Lzd, Cfz, Z	1(5,9)	0(0,0)

Am: Amikacin, Km: Kanamycin, Bdq: Bedaquiline, Hhd: High-dose isoniazid, PAS: Para-amino salicylic acid, Lzd: Linezolid, Cfz: Clofazimine, Z: Pyrazinamide, Lfx: Levofloxacin, Pto: Prothionamide, Mpm-clv: Meropenem–clavulanic acid, Cs: Cycloserine

### Sputum conversion

#### Smear microscopy

**Fig 1:**
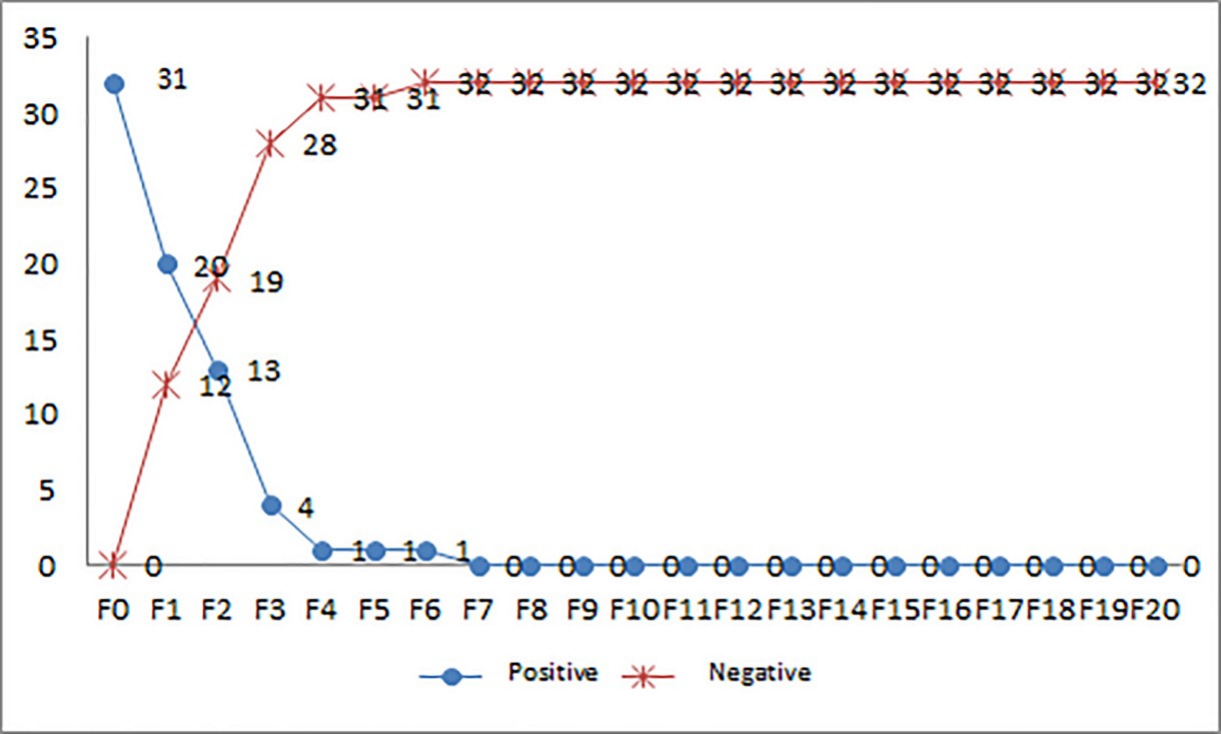
Bacteriological evolution of patients according to the microscopy sputum smears.

Sputum smears were performed as described. The majority were negative at three months. One patient had extrapulmonary tuberculosis (PreXDR Pott disease). The bacteriological conversion was 31.3% at 1 month, 59.4% at 2 months, 87.5% at 3 months, 96.9% at 6 months, 100% at 7 months and 100% at 20 months. The median bacteriological conversion time was 1 (1–6) months. Fig 1 summarizes the bacteriological smear sputum conversion by microscopy.

#### Cultures

**Fig 2:**
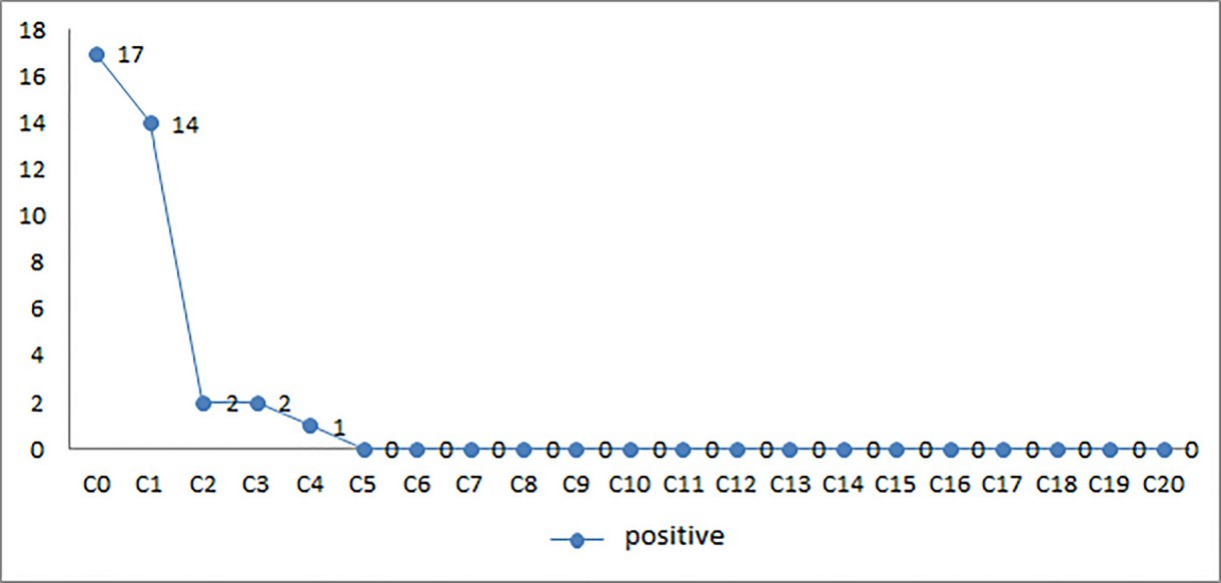
Bacteriological evolution of patients according to cultures.

Seventeen patients had regular culture results. The bacteriological conversion by cultures in these 17 patients was 17.6% at 1 month, 88.2% at 2 months, 88.2% at 3 months, 94.1% at 4 months and 100% at 5 months. The median bacteriological culture conversion time was 2 (1–4) months. Fig 2 summarizes the bacteriological conversion in the culture.

### Study of patient survival: Vital issue

Thirty-two patients were followed for bedaquiline-containing treatment; 15 died (46.8%), and 17 had favorable outcomes (53.2%). Additional RH resistance, HIV-positive status and lung lesions emerged as factors influencing patient survival (Table 5).

**Table 5:** General characteristics according to survival outcomes.

** Variables**	**Alive n = 17**	**Died n = 15**	**P value**
**Resistance Category**			0,378
Pre-XDR _SLID(Am/Km)_	1 (5,9)	3 (20,0)	
Pre-XDR _FQ_	15 (88,2)	10 (66,7)	
XDR	1 (5,9)	2 (13,3)	
RHES			0,463
Resistance	7 (41,2)	5 (33,3)	
Not Known	10 (58,8)	10 (66,7)	
RH			**0,014**
Resistance	4 (23,5)	8 (53,3)	
Not Known	13 (76,5)	7 (46,7)	
HIV Positive			**0,009**
Yes	17 (100,0)	12 (80,0)	
No	0 (0,0)	3 (20,0)	
Lung lesions			**0,041**
<50%	13 (76,5)	6 (40,0)	
≥50%	4 (23,5)	9 (60,0)	
Previous MDR TB			0,589
Yes	5 (29,4)	4 (26,7)	
No	12 (70,6)	11 (73,3)	

### Independent predictors of mortality

A univariate logistic regression was used to identify risk factors associated with mortality (Table 6), and statistical significance was observed for an age ≥40, RH resistance, undernutrition, HIV positivity, severe peripheral neuropathy and optic neuropathy, skin rash and hyperuricemia. A multivariate analysis was subsequently applied to assess the effect of multiple factors on the mortality of patients; five independent factors remained significantly associated with death. These factors included RH resistance, HIV positivity, severe peripheral neuropathy and optic neuropathy, skin rash and lung lesions beyond 50% (Table 6). Skin rash increased the risk of death by 8 times, and severe peripheral neuropathy and lung lesions beyond 50% increased the risk by 7 times.

**Table 6:** Independent predictors of mortality.

**Variables**	**Univariate Analysis**		**Multivariate Analysis**	
	p	HR (95% CI)	p	HRa (95% CI)
Age				
<40 years		**1**		**1**
≥40 years	**0,014**	3,02 (1,89–6,16)	0,395	1,98 (0,41–9,61)
RH				
Not Known		1		1
Resistance	**0,005**	3,08 (2,97–9,77)	**0,011**	2,47 (2,82–7,46)
BMI				
Normal		1		1
Undernutrition	**0,015**	2,99 (1,66–7,70)	0,322	1,97 (0,52–7,53)
HIV positive				
No		1		1
Yes	**0,007**	7,54 (1,74–10,66)	**<0,001**	4,04 (2,73–9,47)
Peripheral Neur and optic				
No		1		1
Yes	**0,006**	5,11 (1,60–16,33)	**0,014**	7,44 (1,50–10,78)
Skin rash				
No		1		1
Yes	**0,007**	5,89 (1,62–20,67)	**0,008**	7,72 (1,70–11,00)
Hyper uricemia				
No		1		1
Yes	**0,012**	2,71 (1,76–9,66)	0,501	1,78 (0,33–9,60)
Lung lesions				
<50%		1		1
≥50%	0,043	2,91 (1,03–8,22)	**0,017**	7,05 (1,42–10,35)

#### Survival analysis

**Fig 3:**
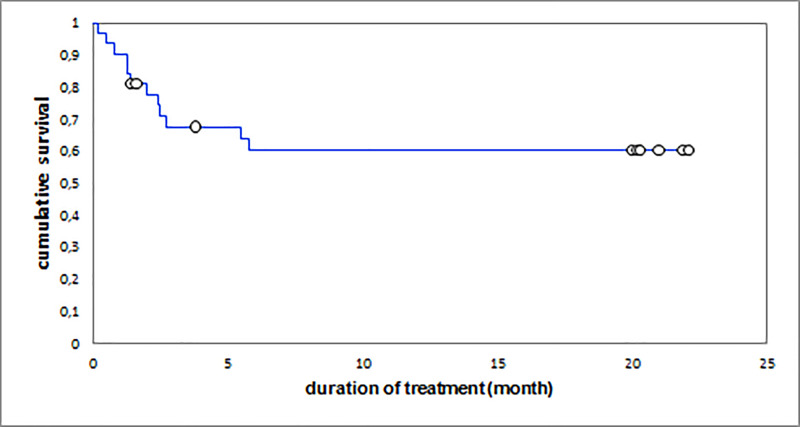
Global survival curve.

**Fig 4:**
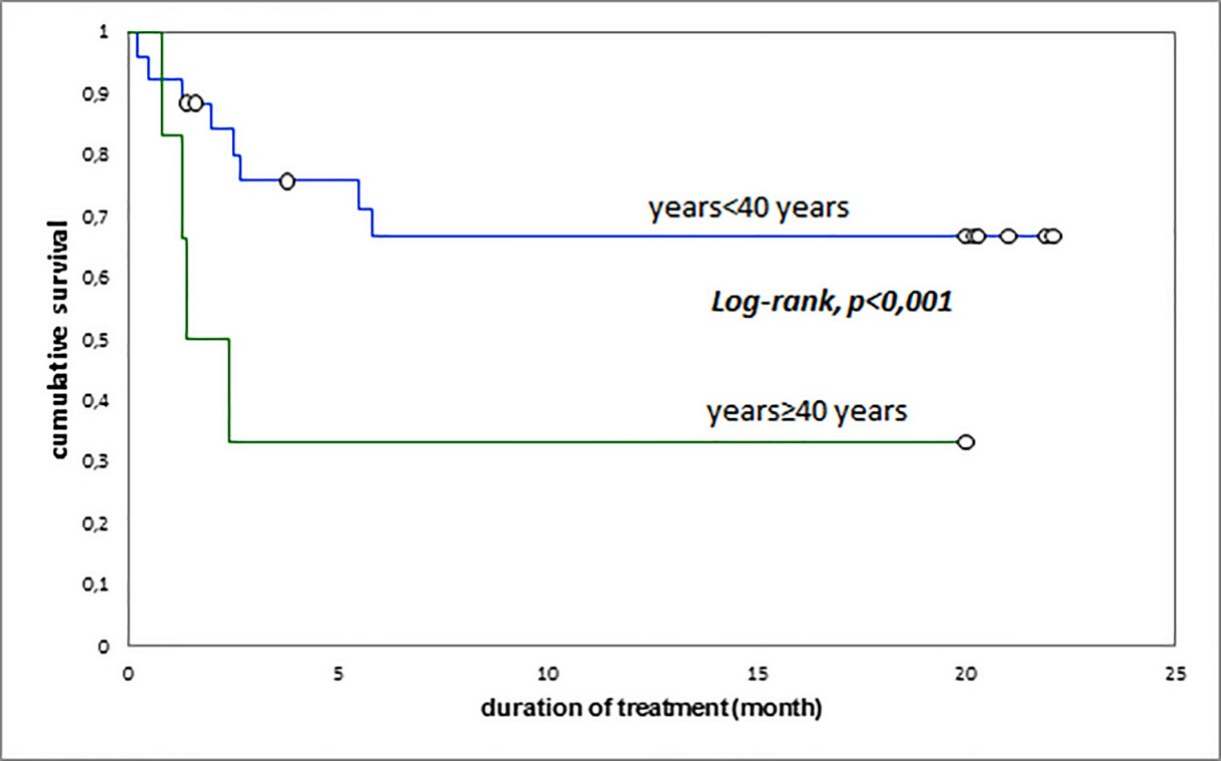
Survival curves of the patients by age.

**Fig 5:**
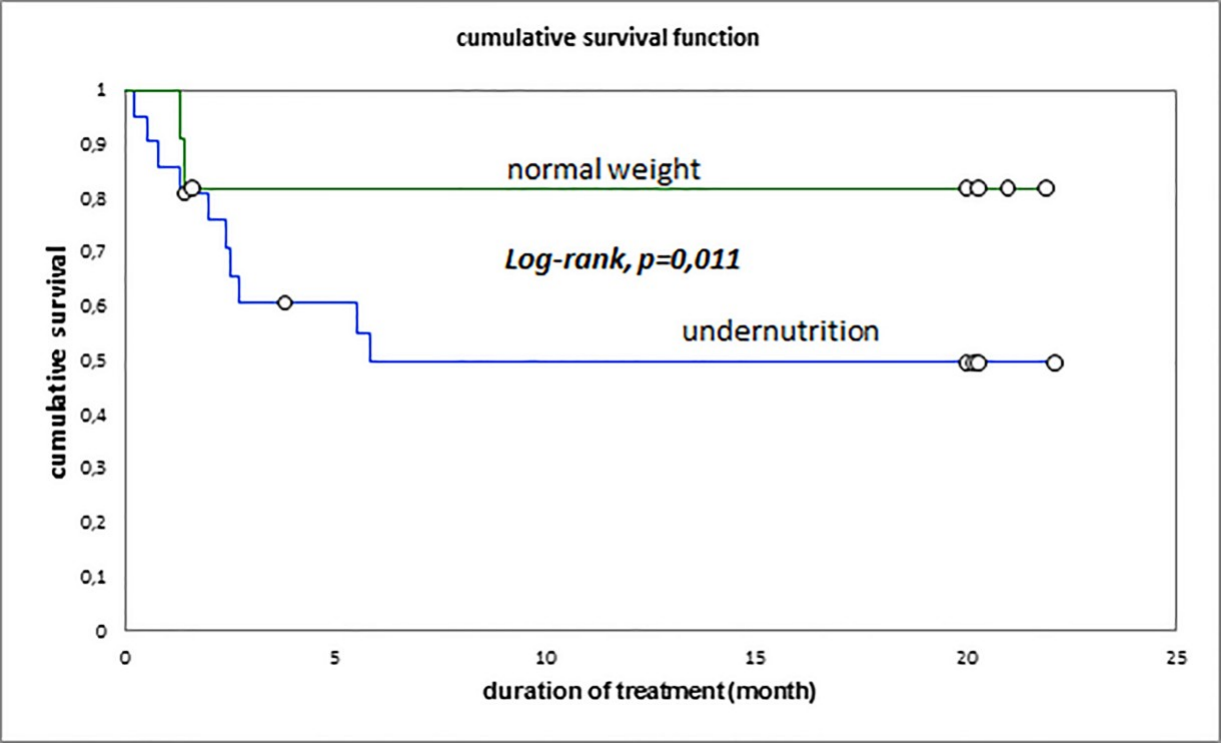
Survival by nutritional status.

The cumulative survival rates were 62.5% at 3 months, 53.1% at 6 months and 53.1% at 20 months. The overall median survival of patients was 5.8 (EIQ: 2.4–20) months; it was 1.6 (EIQ: 1.3–2.5) months in patients who eventually died and 20 (EIQ: 20–20.2) months in patients who were cured (p <0.001) (Fig 1). Undernutrition, age above 40 years, and RH resistance were associated with reduced patient survival (Figs 3–5). The Fig 3 summarize the global survival curve; Fig 4 summarize the survival curves by age and Fig 5 summarize the survival curves by nutritional status.

## Discussion

The treatment of patients with MDR TB, pre-XDR TB or XDR TB involves second-line drugs. These drugs are much more expensive, less effective and have more side effects than first-line TB drugs. The construction of individual treatment regimens for those patients poses several challenges and is complicated by the limited choice of drugs available.

This was the first study in the DRC to describe the experience of managing adverse events of new tuberculosis therapeutics and the outcomes of a large cohort of pre-XDR and XDR TB patients.

The epidemiological profile of the patients in our study (majority were young male patients) is similar to that found in the series of Borissov, Ndjeka and Hewison [17, 32, 33] but different from the series of Peterson in Durban [34].

The majority of patients were previously treated for multidrug-resistant tuberculosis, which suggests that the generalization of drug susceptibility testing for all TB patients should be advocated for as baseline investigations before starting a TB regimen in our country; obtaining an early diagnosis and appropriate treatment before structural lung damage is very important for favorable outcomes. Several countries have adopted this bold strategy with promising results [35–37]. The HIV coinfection rate was low in our series. This corresponds to the prevalence of HIV in our country and is similar to the rate of coinfections in susceptible TB patients in the DRC [38]. Diabetes mellitus was also reported in this study; it is very important to look for this disease and treat it effectively in tuberculosis patients as described in the literature [39, 40].

In the 2015 cohort, only 2 patients out of 21 patients screened were treated with favorable outcome (treatment completed); four patients of that cohort died before treatment. In the 2016 cohort, eleven out of fifteen treated patients died. This was the worst result observed, with only 36% achieving favorable outcomes. Some factors can explain this finding: first, these were patients who had been waiting for treatment since 2015 due to a lack of available drugs; second, those patients had a very poor general condition at the initiation of treatment, and they had very extensive lung lesions on chest radiography with poor prognoses. In the 2017 cohort, a clear improvement was observed with only four patient deaths (73% achieved favorable outcomes). Unfortunately no patient has been declared cured because of the difficulty of regularly performing sputum culture in our country due to lake of funding.

Some cases of sudden death have been noted; hypokalemia was the most likely cause; indeed, the ECGs showed flat T waves corresponding probably to low serum potassium levels. This may be due to the difficulty of measuring serum potassium levels in the hospital, which needs to be performed in another laboratory and probably leads to false normal results due to hemolysis during sample transport. Preventive supplementation with oral potassium and magnesium was introduced in 2016 for all patients with flat T waves.

The addition of high-dose INH in 2015 and 2016 was very poorly tolerated; its subsequent withdrawal from the treatment strategy in 2017 has greatly improved patient tolerance.

The rate of favorable outcomes of pre-XDR and XDR TB patients in the DR Congo increased from 10% in 2015 (WHO TB report 2018) globally to 53.2% in this study after 3 years of experience with bedaquiline; but the result of only 2017 cohort in Kinshasa was 73.3%. These results are better than those declared in the WHO TB report in 2017 and 2018 (globally 30% to 34%) and are also better than those found in the literature of patients treated with similar choices at the same time [20, 41]. However, these results are significantly worse compared to the results obtained by Norbert Ndjeka in South Africa in 2018 (73.0%) and Borisov (72%) in 2017 [17, 35, 38, 42]; In the study presented by Ndjeka et al the use of injectable was not favored [35]. Injectable agents have been associated with increased mortality [43]. This was not seen in our series in 2017; this suggests the possible presence of several mortality factors. The mortality rate in other different cohorts was also lower [17, 38, 42]. News strategy including early diagnosis before lung damage, avaibility of better second line drugs (like the new therapeutic drugs), early management of adverse event and patient support are the key for the better management of these patients. This highlights the need to improve the therapeutic compositions of other new validated second-line drugs, such as delamanid and pretomanid.

The mortality rate remained high in our study. Five independent factors remained significantly associated with death. These factors included RH resistance, HIV positivity, severe peripheral neuropathy and optic neuropathy, skin rash and lung lesions beyond 50%. These factors must be taken into account in the early management of adverse effects with modifications to the previously incriminated second-line drugs; in our study, a serious adverse event caused by linezolid was the main factor associated with the deaths. The literature review reports that the incidence of adverse reactions due to the long-term application of linezolid is high [21, 44, 45]. The role of linezolid is certainly strong in the effectiveness of therapeutic combinations; early management of its adverse effects will allow better compliance. There are currently alternative replacements, such as delamanid, imipenem, clavulanic acid and recently pretomanid [46].

In our study, in the one-year post-treatment follow-up, we found no relapses or failures; this result means that the composition of the bedaquiline-containing treatment was effective and the replacement of poorly tolerated drugs may improve the treatment outcomes. We also did not lose any patients to follow-up; this can be explained by the therapeutic education provided to patients during their hospitalization and especially the monthly financial support for the transport to facilitate travel during the decentralized approach.

In this study, we founds that extensive lung damage was a factor associated with death in pre-XDR/XDR TB patients. This result was also found in MDR-TB patients [39, 40]. The early detection of drug resistance and appropriate timely management of diagnosed patients to prevent extensive lung damage is key to guaranteeing success.

## Conclusion

The new anti-tuberculosis drugs are a real hope for the management of drug resistant tuberculosis patient and other new therapeutic combinations may improve favorable outcomes. However, the improvement of the management strategy including early diagnosis, availability of second-line anti-tuberculosis drugs for a best regimen and better management of adverse event is the key for the future better results.

### Limitation of this study

This work is a retrospective review of the PNT based on hospital monitoring of patients. It is not a planned prospective study.

The strong point of this work is that it highlighted one of the first real-life experiences with bedaquiline and linezolid from pre-XDR and XDR-TB in heavy-burdened Nation
